# Mapping molar shapes on signaling pathways

**DOI:** 10.1371/journal.pcbi.1008436

**Published:** 2020-12-14

**Authors:** Wataru Morita, Naoki Morimoto, Jukka Jernvall

**Affiliations:** 1 Department of Anthropology, National Museum of Nature and Science, Tsukuba, Ibaraki, Japan; 2 Laboratory of Physical Anthropology, Department of Zoology, Graduate School of Science, Kyoto University, Kyoto, Kyoto, Japan; 3 Institute of Biotechnology and Department of Geosciences and Geography, University of Helsinki, Helsinki, Finland; Temple University, UNITED STATES

## Abstract

A major challenge in evolutionary developmental biology is to understand how genetic mutations underlie phenotypic changes. In principle, selective pressures on the phenotype screen the gene pool of the population. Teeth are an excellent model for understanding evolutionary changes in the genotype-phenotype relationship since they exist throughout vertebrates. Genetically modified mice (mutants) with abnormalities in teeth have been used to explore tooth development. The relationship between signaling pathways and molar shape, however, remains elusive due to the high intrinsic complexity of tooth crowns. This hampers our understanding of the extent to which developmental factors explored in mutants explain developmental and phenotypic variation in natural species that represent the consequence of natural selection. Here we combine a novel morphometric method with two kinds of data mining techniques to extract data sets from the three-dimensional surface models of lower first molars: i) machine learning to maximize classification accuracy of 22 mutants, and ii) phylogenetic signal for 31 Murinae species. Major shape variation among mutants is explained by the number of cusps and cusp distribution on a tooth crown. The distribution of mutant mice in morphospace suggests a nonlinear relationship between the signaling pathways and molar shape variation. Comparative analysis of mutants and wild murines reveals that mutant variation overlaps naturally occurring diversity, including more ancestral and derived morphologies. However, taxa with transverse lophs are not fully covered by mutant variation, suggesting experimentally unexplored developmental factors in the evolutionary radiation of Murines.

## Author summary

Teeth are found in almost all vertebrates, and they show many different morphologies. In mammals, especially the cheek teeth or molars are highly diverse in shape, reflecting a vast range of dietary habits and efficiency of occlusion. As teeth are the most durable part of the body, they preserve well in the fossil record. The diversity of molar fossils has been useful in reconstructing the diet and phylogeny of extinct mammals. Genetically modified mice (mutants) show diverse modifications of their molar morphology, but we lack computational tools to test to what extent mutant morphologies account for the natural diversity found in the wild. We developed data mining using machine learning and phylogeny-based methods to analyze three-dimensional molar shapes in mouse mutants and natural species. Although many mutants and species have comparable features, most of the mutant molar variation covers the more evolutionarily ancestral than the more evolutionary derived shapes. Yet to be explored developmental factors may underly the more extreme shapes.

## Introduction

One general challenge in linking the genotype to the phenotype is the multidimensionality of the phenotype. Especially when comparisons include both evolutionary diversity and experimentally produced variants, methods need to account for both small and large differences in the phenotype. Here we explore landmark-free morphometric mapping approaches to examine variation in the mammalian dentition. Dentitions provide many opportunities for the linking of evolutionary transformations with experimental evidence on the genetic control of development. Among jawed vertebrates, mammals show the highest morphological diversity in tooth shape [1,2]. Especially molar teeth show a high diversity of shapes, which is closely associated with different kinds of dietary adaptations among mammalian species [3–6]. Due to the preponderance of teeth in the fossil record, numerous studies have examined dental characters such as cusp and loph arrangement on occlusal surfaces of the molar teeth. Consequently, mammalian molar tooth morphology has played a central role in species identification and reconstruction of phylogeny and diet [6–9]. Concomitantly, tooth development is becoming increasingly better understood. Developmental studies have unveiled when, where, and what kind of genes are expressed and how they interact with each other [10,11]. More than 200 genes have been identified to be dynamically expressed during tooth development. Many of these genes belong to transforming growth factor β (Tgfβ), fibroblast growth factor (Fgf), sonic hedgehog (Shh), and Wnt signaling pathways that are required repeatedly during tooth development.

Much of the experimental data about the role of the different genes in tooth development come from studies using laboratory mice (*Mus musculus*). Mice belong to the subfamily Murinae (murines) of the family Muridae. Murines are one of the most diverse mammal taxa, which has been proposed to be partly due to the evolution of novel molar morphologies contributing to the colonization of a broad spectrum of diets and habitats [12].

Many genetically modified mice have been reported to have distinctive tooth phenotypes [13–16]. Genetic mutations typically simplify crown morphology, and large increases in complexity appear to require the adjustment of multiple signaling pathways simultaneously [17]. More detailed analyses have shown that tooth characters present in stem murines can be reproduced by tinkering with signaling, especially with that of ectodysplasin (Eda), a diffusible tumor necrosis factor (TNF) family protein [18–20].

Tooth shape can be considered an archive of phenotypic variation, encompassing the past, present, and laboratory-derived populations. Examining all these data together, a key question related to evolution is the likelihood of different morphological transformations. Whereas the effects of signaling pathways on tooth phenotype have been characterized in individual cases [13,14,21,22], we lack comprehensive analyses of mutant morphologies in the context of evolutionary diversity. Furthermore, it remains to be explored how different signaling pathways may coinfluence dental characters. In this context, a problem that has hampered quantification of mutant and evolutionary phenotypes is the complexity of the molar morphology itself and the extensive changes that gene inactivations can cause on the morphology. Since it is difficult to assess how and to what extent crown variations differ from each other, the effect of signaling pathways on molar morphology has not been thoroughly examined. The complexity thus remains a challenge for interpreting the effect of gain and loss of function in gene expression on the shape and inferring acquisition of novel characters involved in morphological evolution.

**Fig 1:**
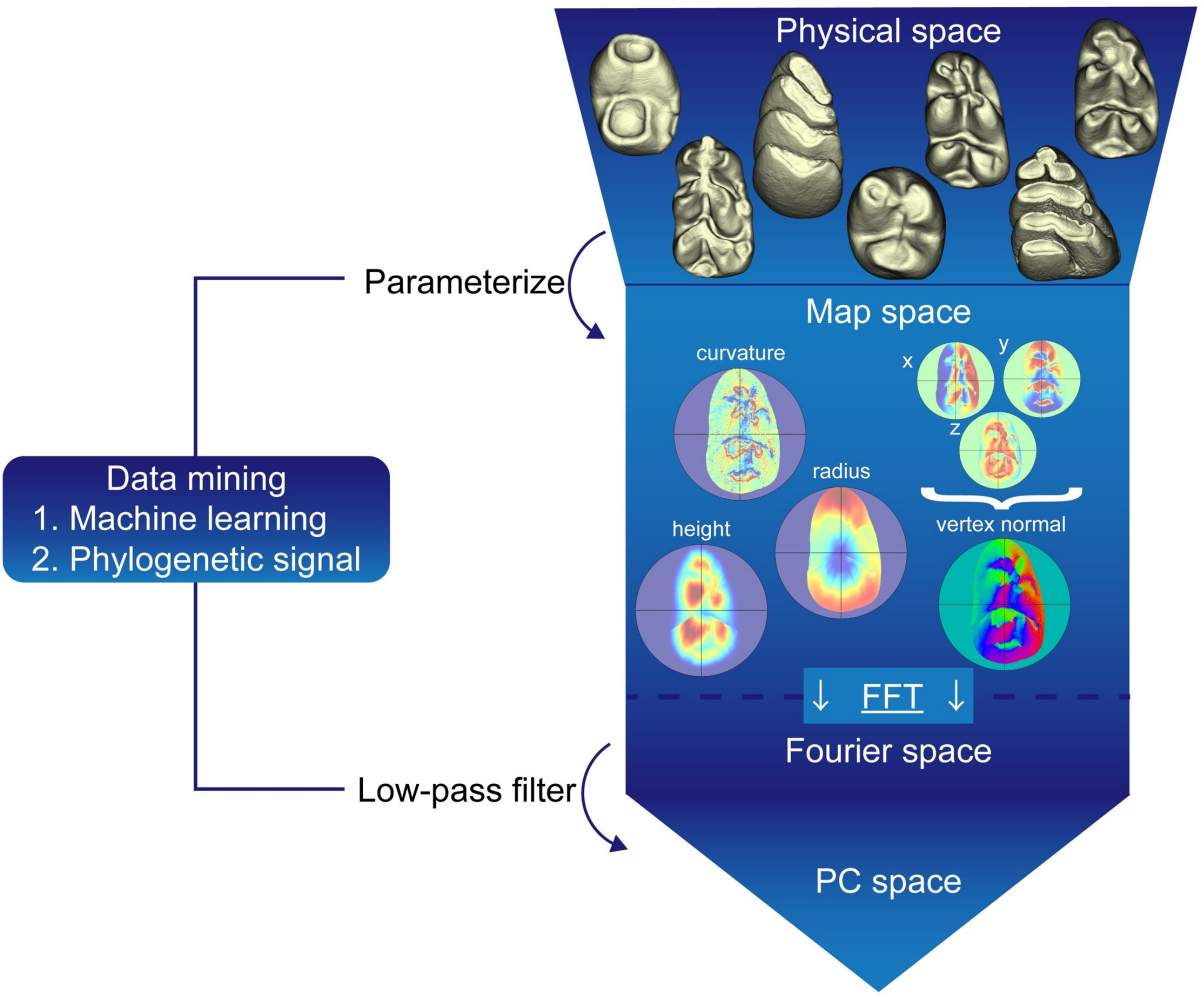
Outline of procedures for morphometric mapping with data mining. 3D-reconstructions of diverse tooth shapes are turned into different morphometric maps that parameterize distinct aspects of the morphology, such as surface curvature, height from the cervix, radius from the center of the tooth crown, and vertex normal. All maps are converted with Fast Fourier Transformation (FFT) for low-pass filtering and finally analyzed by Principal Component Analysis (PCA). Two data mining schemes, machine learning and phylogenetic signal, are performed to select the combination of morphometric parameters and the size of low-pass filtering depending on the composition of the sample.

Here we compare molar shape variation in 31 murine species and 22 mouse mutants using a novel integrated scheme of morphometric mapping (MM), which combines a landmark-free approach for feature extractions with two data mining techniques, machine learning and phylogenetic signal [23] (Fig 1 and S1 Text). This method permits to quantify the complicated morphologies by a set of densely sampling morphometric parameters. While various kinds of morphometric tools and algorithms have been developed over the decade [24–28], it has remained unclear how to select the appropriate variables according to research objective (but see ref. [29]). As a solution to this problem, we introduced two data mining techniques to select appropriate data set which meet the following objectives: i) machine learning for maximizing classification accuracy of mutant mice to clarify the relationship between signaling pathway and molar shape variation and ii) phylogenetic signal that reflects phyletic relationship adequately to place both mutant mice and murine species into the same morphospace, thereby allowing the estimation of the evolutionary coverage of pathway modifications.

## Results

### Machine learning approach to analyze molar shapes of mutant mice

**Fig 2:**
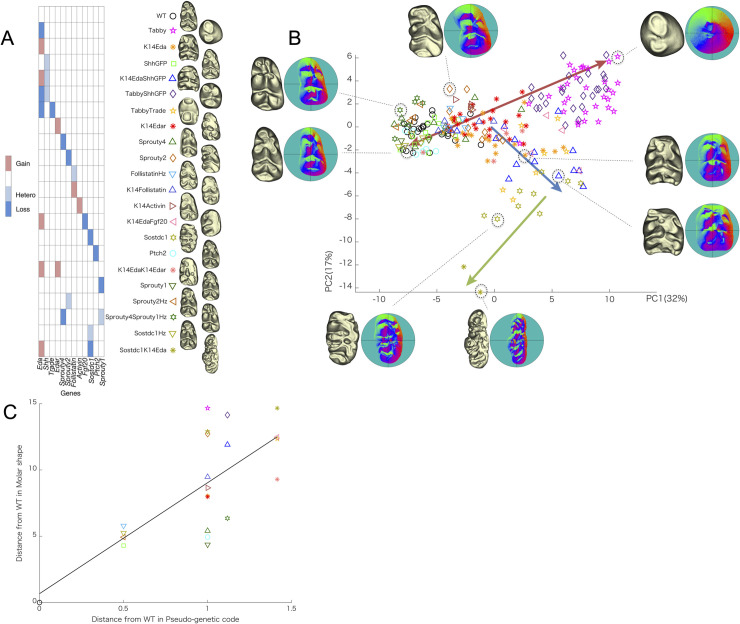
Molar shape variation in 22 mutant strains. (A) Pseudo-genetic code for mutant strains with 3D models of the teeth (symbols correspond to PC plot in B). (B) Shape variation depicted on the first two principal axes of between-group PCA on vertex normal vector with the first six sets of Fourier coefficients. 3D model and corresponding vertex normal map of marked specimens are provided to illustrate shape variation. Red bidirectional arrow shows shape variation associated with the number of cusps. Blue arrow exhibits shape change towards parallel distributed cusps and increased lophedness. Green arrow represents shape change towards a fusion of two molar configurations. (C) The difference between the pseudo-genetic code from WT (x-axis) and phenetic distance from WT (y-axis) shows increasing phenotypic change with genetic change.

Our machine learning approach included steps to parameterize the lower first molar (LM1) of 22 mutant strains representing wild type (WT) with multiple metrics depicting their morphology (Figs 1 and 2A). Here LM1 was chosen because it is the most commonly studied tooth in developmental biology research. Specifically, the three-dimensional (3D) models of the sample (Fig 2A) were parameterized with 4 morphometric parameters (Fig 1): surface curvature, height from the cervix, radius from the centroid of cervical line, and vertex normal that represents the direction of each vertex as a unit vector in three dimensions (x, y, and z variables, respectively). Before performing multivariate analysis, each morphometric map, i.e., the matrix of morphometric variable, was converted into a set of coefficients by Fourier transformation for low-pass filtering. This procedure affects the scale of detail that is used in the subsequent analyses. We applied feature selection algorithms by utilizing machine learning to pick the combination of morphometric variables and the size of low-pass filtering to optimize the classification of the 22 mutants. To obtain a relatively broad range of classifications, we compared seven types of map combinations by adjusting filter size in seven different classification models (five basic learners: decision tree, linear, discriminant, support vector machine, and k-nearest neighbor). Cross-validation was used to evaluate the performance of each model with mean classification loss where data was randomly partitioned into ten subsets, and one subset was used to validate the model trained using the remaining subsets. Five models showed comparable accuracy (accuracy = 1 – averaging cross-validation classification loss) of the best classifiers (79.1%) (S1 Fig and S2 Table). Among them, multivariate analysis was performed using the most parsimonious model in which vertex normal with relatively small (size = 6) low-pass filter were used as the “best” set of variables for the principal component analyses (PCA).

The PCA was performed on the mutant means (that is, between-group PCA; Fig 2B), with the first two components accounting for 32% and 17% of the total variance, respectively. Examining the PCA shows that shape variation along PC1 captures the difference due to the number of cusps. The positive and negative extremes along PC1 correspond to *Tabby* and WT, respectively (Fig 2B). While the former has only 2 or 3 cusps with an oval outline, the latter has a wedged outline. The PC2 captures variation in the outline shape of the tooth, as also relative size and distribution of cusps. Overall, specimens of positive PC2 possess large cusps relative to the crown surface. Most of the teeth from K14*Eda* and K14*EdaShhGFP* are located in the lower middle, and their small cusps relative to the crown surface are distributed in parallel with a rectangular outline. This cusp distribution leads to an increase in both transverse and longitudinal lophedness (crests connecting adjacent cusps). Shape change towards the negative extreme of PC2 eventually reaches a fused m1 and m2 configuration in double mutants of *Sostdc1* knockout K14*Eda*.

### Phenetic distance approximates the genetic distance of the mutants

Because our data set included a different combination of mutants (Fig 2A), including null mutants that have been crossed with overexpression mice for another gene, we approximate the cumulative effects of these genetic mutations. This was calculated as a 'pseudo-genetic distance' of the mutants from WT mice (Materials and Methods). The distance of each mouse strain from the WT was assumed to be the sum of individual mutations. For example, a mouse mutant that is homozygous for one gene and heterozygous for another gene, would have a distance of 1.5 from the WT. Similarly, we calculated the phenetic distance between the mutants and WT teeth as Euclidean distance in morphospace. The two distance matrices show an overall agreement (Fig 2C, r = 0.73, p < 0.001), suggesting that larger changes in signaling result in larger changes in the phenotype. However, as the deviation from the regression line is large when more than one gene is adjusted, the effect cannot be considered simple (Fig 2C), underscoring the multidimensionality of the phenotypes (Fig 2B).

### Molar shapes of wild murines partially overlap the mutant phenotypes

To explore links between developmental modification and evolutionary patterns in molar morphology, we examined how the mutant mice and wild murines are distributed in a morphospace.

Starting with wild murines, shape variables were selected that maximize the phyletic information among the species. Specifically, to select the shape variables that best explain the phylogenetic relationships, we used the multivariate K statistic as the data mining criterion [30]. K statistic provides a statistical measure of the phylogenetic signal relative to expectations under the Brownian motion model of evolution [31]. When K is equal to 1.0, multivariate phenotypic traits analyzed are assumed to evolve at a neutral rate, and its phenotypic variance is proportional to genetic distance. We used 31 wild murine species for the analysis (S3 Table), and their phylogenetic relationships were constructed following Steppan and Schenk [32]. Using the same scheme as in machine learning, we calculated K statistic from various combinations of morphometric variables and the size of low-pass filtering (S2 Fig). The analyses show that K values are lower than 1.0 in all the models considered, the variable combination 'vertex normal with radius' (Nxyzr) and 'low-pass filter size 2' (S4 Table) providing the highest value (K = 0.704). Therefore, closely related species are more different from each other than expected under the Brownian motion model. Because the phenotypic divergence of molars is indicative of ecology, we analyzed the effect of diet on molar shape variation using phylogenetic generalized least squares (PGLS) with the variables providing the highest K value. The result shows that the effect of diet is significant (S5 Table), suggesting ecology in driving murine molar evolution [6].

**Fig 3:**
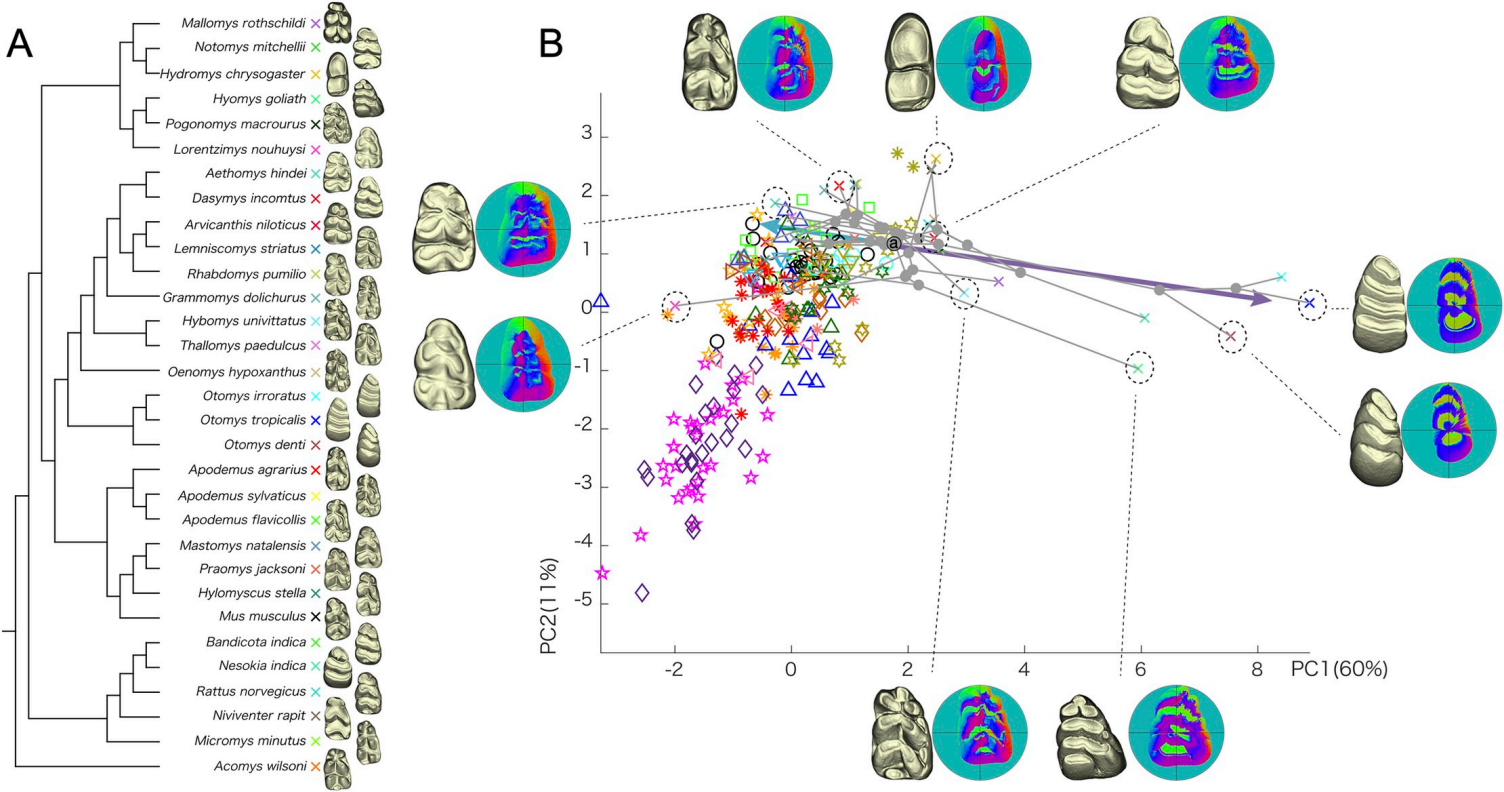
Molar shape variation of wild Murinae species. (A) Phylogenetic tree of murines used for the analysis. *Acomys wilsoni* is included as an outgroup (symbols correspond to PC plot in B). Examples of a 3D model for each taxon are shown. (B) Shape variation depicted on the first two principal axes of between-group PCA on vertex normal vector and radius with the first two sets of Fourier coefficients. Morphometric map representation is the same as Fig 2. Blue−green arrow signifies shape change associated with a wedged outline and relatively large six cusps on a crown. Purple arrow captures shape change towards lamellation. Legends for mutants are the same as in Fig 2. Circled ‘a’ represents the estimated last common ancestor of murines analyzed.

Next, to examine the molars of the mutant mice in the context of murine species, we performed a PCA combining the two datasets. This was done using the model showing the highest K value for murine species (K = 0.704 with Nxyzr and low-pass filter size 2, S4 Table). These variables were extracted from both the mutants and the wild species, and a morphospace was generated using PCA (using the covariance matrix, Fig 3B). The phylogenetic tree (Fig 3A) was projected onto morphospace by reconstructing hypothetical ancestral morphologies (that is, internal nodes) using squared-change parsimony [33]. When the two PC spaces are compared (Figs 2B and 3B), they appear roughly similar since the distance matrices of the first two PC scores are highly correlated (r = 0.93, p < 0.001). Thus, shape space defined by mutants and wild murine variation is largely comparable to that of mutants, although the order of the first two PC axes is switched with each other between these two PCAs.

Examination of the PC shows that wild murines exhibit two distinct shape trends from the estimated common ancestor of all Murinae rodents. The first is towards the negative PC1 direction, which shows a wedged outline with six relatively large main cusps and lacking accessory cusps, such as in the genus *Mus* itself. The second is towards the positive PC1 direction, which exhibits the most notable shape variation, that is, lamellation of cusps seen in *Otomys*. This variation is not accounted for by the mutant phenotypes. Conversely, mutant phenotypes characterized by a small number of cusps and rounded tooth shape (e.g., *Tabby* mutants, Fig 2) do not have corresponding mouse taxa. Overall, whereas there is considerable overlap between the mouse mutant and murine species morphologies, both groups also exhibit distinct phenotypic variants.

## Discussion

### Relationship between signaling pathways and molar shape

Our results of machine learning showed that the combination of vertex normal with a relatively small number of Fourier coefficients for low-pass filtering was efficient for the purpose of classification of mutant molars. The vertex normal quantifies detailed (i.e., local scale) morphology of the crown. On the other hand, a small number of Fourier coefficients permit to capture global feature of the respective parameter. Thus, in the data set we used, the global features of the local scale morphology reflected the genetic differences between mutants. How can this be related to development? It is plausible that the global features capturing small scale morphology (here, the vertex normal) are related to that of cusp patterning that is largely determined at the morphogenetic phase of tooth development. This result no doubt reflects the large changes that can be caused by mutations (Fig 2A).

Although mutants show a quite broad spectrum in dental morphology [18,34], their morphological variation can be described largely as a combination pattern of the number, relative size, and distribution of cusps, and outline shape of molar crowns (Fig 2). Most importantly, the relationship between signaling pathways and molar shape is not necessarily linear. For example, mutant strains related to Eda signaling (*Tabby* and K14*Eda*) and WT are not aligned on a straight line in morphospace. Additionally, *Sostdc1* heterozygous mutant mice are not located in the middle between WT and *Sostdc1* null mice. Consequently, the correlation between phenetic distance matrix data and the pseudo-genetic distance matrix was moderate (r = 0.48). Nevertheless, disparity from WT between pseudo-genetic code and molar shape shows relatively high correlation and R-squared coefficient (r = 0.73, R^2^ = 0.53; Fig 2C). This may stem from the fact that shape changes in heterozygous strains in subtle ways, and the extent of shape change is rendered unstable when more than one genetic loci are adjusted in unison. Signaling pathways expressed in tooth development are iteratively used during morphogenesis, and small effects may have cascading effects over development.

### Evo-devo perspective on molar shape variation in murine rodents and mouse mutants

Using the data set composed of vertex normal and radius with the first two sets of Fourier coefficient that provides the highest phylogenetic signal, we compared the shape variation of wild murines with mutants. The inferred last common ancestor of murines is located close to *Sostdc1* knockout specimens, suggesting they share complex molar morphology. The Murinae supposedly originated in the Middle Miocene of southern Asia around 14 million years ago [35,36]. The fossils assigned to early murines exhibit fairly complicated crown structure with not only six cusps but also several accessory cusps [37], which is largely consistent with the estimated common ancestor in our analysis (Fig 3). Although we used a dataset that reflects phylogeny among the specimens, the phylogenetic signal in our data was lower than expected under neutral evolution. This indicates prevalent convergent evolution in murine radiations. Selective pressures on dietary adaptations are a likely explanation for some of the convergent morphologies (S5 Table [6]). These similarities include complex crown morphologies that also include longitudinal crest (called stephanodonty), features also present in some of the mouse mutants [19]. Nevertheless, considering the relatively extensive phenotypic changes produced by single gene mutations in our data (Fig 3B), convergent morphologies do not necessarily imply convergent adaptation.

Whereas our results show an overlap between wild murine diversity and mutant shape variation, several taxa are out of the mutant range (S3 Fig). This indicates that the morphological diversity found in wild murines is not fully explained by the developmental factors in the currently known mutants. In other words, 'hopeful monsters' produced in the laboratory are not a match for the 'wild beast' produced by evolution. Specifically, our PC space made up of mutants and wild species is a type of theoretical morphospace [38] and can suggest boundaries of developmental or phylogenetic constraints. The simplified morphology of *Tabby*, the null mutant of *Eda*, does not have an equivalent in the wild species in our sample. This does not exclude the possibility that in specific selective regimes, a comparable morphology could be attained in evolution. Indeed, *Rhynchomys* species with a high proportion of worms in their diet show a reduction in their dental complexity reminiscent of *Tabby* teeth [39].

The lamellation, which is captured as disparate shape variation among wild murines, is not realized in the studied mutants. This is suggestive that some additional, yet to be discovered factors may regulate the formation of these tightly packed lateral crests. Although the lateral placement of cusps and stronger development of transverse crests have been reported for Eda pathway overactivation [17], these morphologies do not approach the extreme lamellation seen in *Otomys*. One possibility is that strong selective pressures related to a fibrous diet have selected both the Eda pathway and other pathways is regulating lamellation. Yet another possibility is that regulation of lateral cusp configuration can be partly outsourced to the adjacent jawbone [40].

Overall, our approach overcomes the challenge of studying shapes that lack homologous landmarks among disparate shapes. This, in turn, allowed us to compare disparate mutants and contrast these shapes to real species. These data and analyses help to identify areas where we still lack experimental evidence on the regulation of the phenotype. Conversely, developmental data can be used to project the kind of phenotypic variants that are likely to evolve. Combining phenotypic and genotypic information based on comparative data in light of naturally selected variation will provide us with a further understanding of evolutionary modification of development.

## Materials and methods

### Mutant and wild murine samples used in the study

Our total mutant sample (N = 235) consists of 22 strains (including wild type) related to 12 signal transduction genes (S1 Table). Each strain represents null (knockout), heterozygote-null, overexpression under keratin-14 promoter, or a combination of two different gene modifications. Mice were maintained on a mixed genetic background, and littermates were used as wild type. All mutant strains are stored at the University of Helsinki. Comparative data set of Murinae, a subfamily of Muridae, consists of 31 species, including *Acomys wilsoni* (Deomyinae) as an outgroup (S3 Table).

All the specimens were micro-CT scanned at the Department of Physics, University of Helsinki, Finland, using SkyScan 1272, Bruker with voxel resolution of 10 μm. The 3D-reconstructions were made using the software Amira 6.4 (Thermo Fisher Scientific, Waltham, Massachusetts, USA).

### Morphometric mapping with data mining

We used a landmark-free morphometric mapping approach, which is suitable for morphologically highly complicated and extremely variable teeth used in this study. The workflow of this method is represented in Fig 1 and S1 Text. Prior to parametrize 3D molar shape with morphometric variables, each 3D surface model was placed in the Cartesian coordinate system following Morita et al. [23]. Briefly, the cervical line was manually digitized on the surface model, and the least-squares plane of the outline was computed. Each molar was positioned so that the cervical plane was horizontal to xy-plane (z = 0). The molar was centered on matching with the centroid of this coordinate system, followed by shifting-down of the 3D model along the z-axis up to the upper 80% of tooth crown height (from the highest cusp tip to the cervical plane) being above xy-plane. This procedure ensures that the analyses are done on the cuspal morphology of the teeth. Next, the molar surface was sectioned by 300 vertical planes radiating from the z-axis, perpendicular to the basal (x−y) plane, and cross-sectioning the molar in 300 equiangular sections (L = 300). In each vertical cross-section, 300 points were sampled along the intersection of the vertical section with the EDJ surface, running from the z-axis to the outer intersection at z = 0. This was done in 300 equidistant intervals (K = 300). For each of the 300 sampled points along the 300 equiangular sections, the following morphometric parameters were recorded: surface curvature (c), height from the basal plane (h), horizontal distance, i.e., radius, from the centroid (r), and vertex normal that represents the direction of the local area in 3D as a unit vector (Nxyz). The morphometric data of each molar were thus represented as K × L matrices (K = 300, L = 300) for each of the four parameters of six variables (c, h, r, and Nxyz). The morphometric variables were mapped onto a polar coordinate system (d, θ). Here, d denotes the normalized position along each cross-sectional outline (d = 0→1: center→outer crown base), and θ denotes the anatomical direction (θ = 0°→360°: buccal→mesial→lingual→distal→buccal). The 3D morphology of the crown surface was thus visualized in the form of two-dimensional morphometric maps M (d, θ). These morphometric maps are shown as false-color maps. Each element of the vertex normal vector (x, y, z) is assigned to an RGB value, respectively, and integrated into a map. To facilitate visual inspection, morphometric maps are reconstructed to represent the outline at the cervix by padding the background according to relative length at the cervix. The effects of scaling were corrected by normalization of the variables c, h, and r using centroid size (CS, the square root of the summed squared distances calculated from h and r variables). This approach is analogous to normalization by centroid size in standard geometric morphometrics [41]. Each row of the K × L matrix for each specimen was sequentially weighted by a concentrically subdivided area with radius 1 and constant internal angle (= 1/L) that was equidistantly sectioned (= 1/K). Each specimen was preoriented according to the anatomical direction. Then, 2D-Fourier transforms F(M_i_) of all M_i_ (i = 1, 2, …, n) were calculated (M had natural periodicity in θ). This resulted in K × L sets of Fourier coefficients representing a specimen’s shape of the EDJ surface as a point in the multidimensional Fourier space. Differences due to orientation were corrected, limiting the freedom of rotation to only around the z-axis. This optimal fitting was achieved by iteratively minimizing inter-specimen distance in Fourier space through rotation around θ (z-axis).

After all specimens were aligned by the optimal fitting, we carried out two data mining techniques: i) machine learning, and ii) phylogenetic signal, to select a combination of morphometric variables and the number of a set of Fourier coefficients (i.e., the size of low-pass filtering in Fourier space) to reduce the number of variables relative to the number of specimens and to focus on the major patterns of shape variation.

In the first scheme, machine learning was used to maximize the classification accuracy of 22 mutant strains. We trained multiclass error-correcting output codes (ECOC) model [42], using five types of leaners: decision tree, linear classification, discriminant analysis, support vector machine (SVM), and k-nearest neighbors (number of neighbors was set to 1, 3, and 5 with neighborhood defined by Euclidean distance). 10-fold cross-validation was used as a measure of model accuracy with the seven classification models described above. We compared seven combinations of morphometric variables with adjusting the size of low-pass filtering from 1 to 50: chr, c, hr, Nxyz, Nxy, Nxyzr, chrNxyz.

For the second scheme, the multivariate K statistic was used to maximize the phylogenetic signal among the sample implemented with the R package geomorph [30, 43]. The phylogeny of murine rodents was modified from the molecular phylogeny of Steppan and Schenk [32], which is based on multiple nuclear and mitochondrial markers and included multiple fossil calibrations. *Aethomys hindei*, *Apodemus sylvaticus*, and *Notomys mitchellii* were not in Steppan and Schenk (2017), and we estimated the position of these taxa at the generic level with Mesquite v. 3.05 [44]. We compared the same seven types of map combinations with adjustment of low-pass filter size from 1 to 100.

### Multivariate analysis

After data mining procedures, we conducted two between-group principal component analysis (PCA). We performed PCA on the data set of only mutants according to the result of machine learning, and that of both mutants and wild murines based on the phylogenetic signal, respectively. The phylogenetic tree was projected onto the latter PC space with reconstructed internal nodes (the position of ancestral states) using the maximum likelihood method for continuous data implemented with the R package phytools [33,45]. Group-mean molar shapes of mutants were used to evaluate a between-population phenetic distance matrix. The scheme for pseudo-genetic coding is based on the expression of each signaling: loss, 0; hetero, 0.5; gain, 2; normal, 1. The ‘pseudo-genetic distance’ from WT was defined as the disparity from the normal based on this coding system. The disparity from the wild type was evaluated in both phenetic and pseudo-genetic data to examine the phenetic-genetic relationship. Furthermore, to evaluate the correlation between molar shape and pseudo-genetic distance matrices for mutants, the Mantel test was used. All parametrization and calculations for MM were performed in MATLAB 9.5 (MathWorks, USA). To evaluate the effect of diet on molar shape variation, we used phylogenetic generalized least squares (PGLS) with R packages geomorph [43]. The murine species were placed into four dietary categories (generalist, herbivore, insectivore, and frugivore) obtained primarily from the recent summary of the literature [46,47] where they emphasized on the primary resource in a given diet, and also by consulting the cited primary literature [48,49].

## Supporting information

S1 Text A pipeline of data acquisition procedure for morphometric mapping. (DOCX)

S1 Fig Results of machine learning of seven types of probabilistic classification models for seven map variable combinations. (A) chr, surface curvature, height, and radius; (B) c, surface curvature; (C) hr, height and radius; (D) Nxyz, vertex normal; (E) Nxy, two elements of vertex normal; (F) Nxyzr, vertex normal and radius; (G) chrNxyz, surface curvature, height, radius, and vertex normal. Size of low-pass filter is equal to the number of the set of Fourier coefficients for the analysis. SVM, support vector machine. KNear, k-nearest neighbor (number of neighbors was set to 1, 3, and 5).
(TIF)

S2 Fig Comparison of phylogenetic signal in seven map combinations. chr, surface curvature, height, and radius; Nxyz, vertex normal; Nxy, two elements of vertex normal; Nxyzr, vertex normal and radius; c, surface curvature; hr, height and radius; chrNxyz, surface curvature, height, radius, and vertex normal. Size of low-pass filter is equal to the number of the set of Fourier coefficients for the analysis.
(TIF)

S3 Fig Comparison in molar shape variation between mutants and wild murine. (A) Phylogenetic tree of murines in this study. Taxa locating outside of the mutant range in between-group PC (PC) space are colored with blue otherwise done in magenta. (B) Convex hulls enclose the range of variation of mutants (magenta) and wild murine species (blue) in the first two principal axes of PCA.
(TIF)

S1 Table Sample of 22 mutant strains. (DOCX)

S2 Table Top twenty probabilistic classification models in machine learning for 22 mutant strains. Nxyz, vertex normal; Nxy, two elements of vertex normal; Nxyzr, vertex normal and radius. SVM, Support vector machine.
(DOCX)

S3 Table Sample for wild murine species analysis. *Collection source: §Naturhistorica Riksmuseet Stockholm; †Luonnontieteellinen Museo Helsinki; ‡Institute of Biotechnology, University of Helsinki. Geographical distribution is based on [22,23]. Body mass (mean weight in g) is cited from [24]. Ave. Centroid size is calculated from three-dimensional surface model.
(DOCX)

S4 Table Top twenty models of phylogenetic signal. hr, height and radius; Nxyz, vertex normal; Nxy, two elements of vertex normal; Nxyzr, vertex normal and radius.
(DOCX)

S5 Table Results of phylogenetic generalized least squares (PGLS) on molar shape variation of 31 wild murine species. (DOCX)
