# Supplementary material for: Mapping molar shapes on signaling pathways
Source: PLoS Comput Biol. 2020 Dec 14;16(12):e1008436. doi: 10.1371/journal.pcbi.1008436 (PMC7735603; doi:10.1371/journal.pcbi.1008436)
Supplement: S1 Text — (DOCX) [file pcbi.1008436.s001.docx]

1. All the specimens are microCT scanned.
2. Reconstruct the 3D surface model.
3. Convert a 3D model into 2D map images using morphometric mapping method:
   - - - Parameterize with several morphometric variables: surface curvature, height, radius, vertex normal, enamel thickness, Dirichlet normal energy, etc.
4. Execute optimal fitting for all the specimens.
5. Convert 2D map images constructed by each morphometric variable into a set of Fourier coefficients
6. Execute data mining across several combinations of morphometric variables (maps) with stepwise addition of the number of Fourier coefficients from the low-frequency domain (low-pass filtering). The choice of data mining criterion depends on research design:
   1. Machine learning for classification problem: maximize classification accuracy using one or more sorts of learners, such as decision tree, k-nearest neighbors, support vector machine, etc.
   2. Phylogenetic signal for phyletic analysis: maximize phylogenetic signal referring to a molecular phylogenetic tree. Several sorts of phylogenetic signals are available [1].
7. Execute a multivariate analysis with the data set selected by the data mining procedure.

**S1 Text.** A pipeline of data acquisition procedure for morphometric mapping.

**References**

1. Adams DC, Collyer ML. Multivariate Phylogenetic Comparative Methods: Evaluations, Comparisons, and Recommendations. Syst Biol. 2018;67(1):14-31.
