# Supplementary figures and images for: Mapping molar shapes on signaling pathways
Source: PLoS Comput Biol. 2020 Dec 14;16(12):e1008436. doi: 10.1371/journal.pcbi.1008436 (PMC7735603; doi:10.1371/journal.pcbi.1008436)

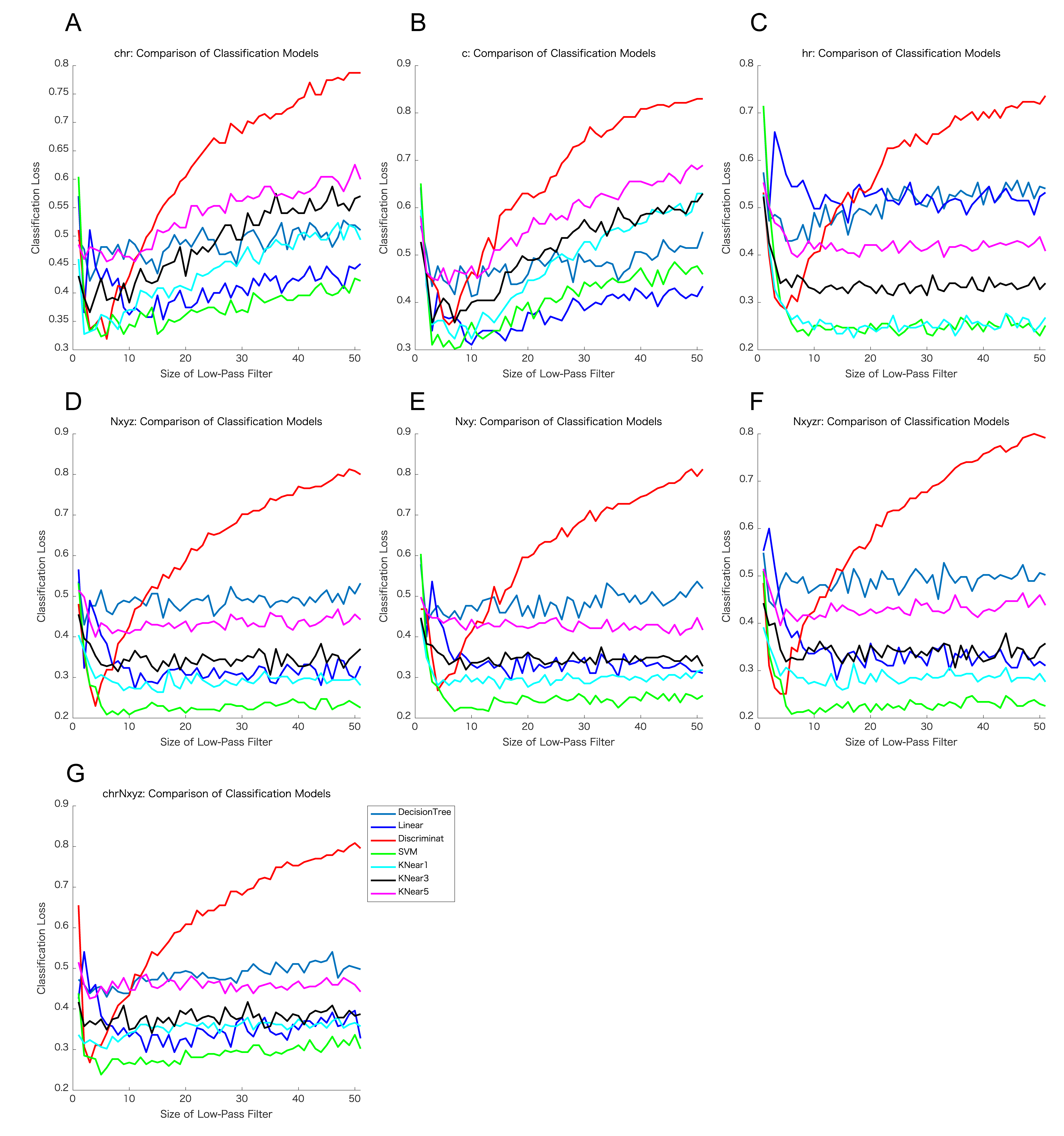

Supplement: S1 Fig — (A) chr, surface curvature, height, and radius; (B) c, surface curvature; (C) hr, height and radius; (D) Nxyz, vertex normal; (E) Nxy, two elements of vertex normal; (F) Nxyzr, vertex normal and radius; (G) chrNxyz, surface curvature, height, radius, and vertex normal. Size of low-pass filter is equal to the number of the set of Fourier coefficients for the analysis. SVM, support vector machine. KNear, k-nearest neighbor (number of neighbors was set to 1, 3, and 5). (TIF) [file pcbi.1008436.s002.tif]

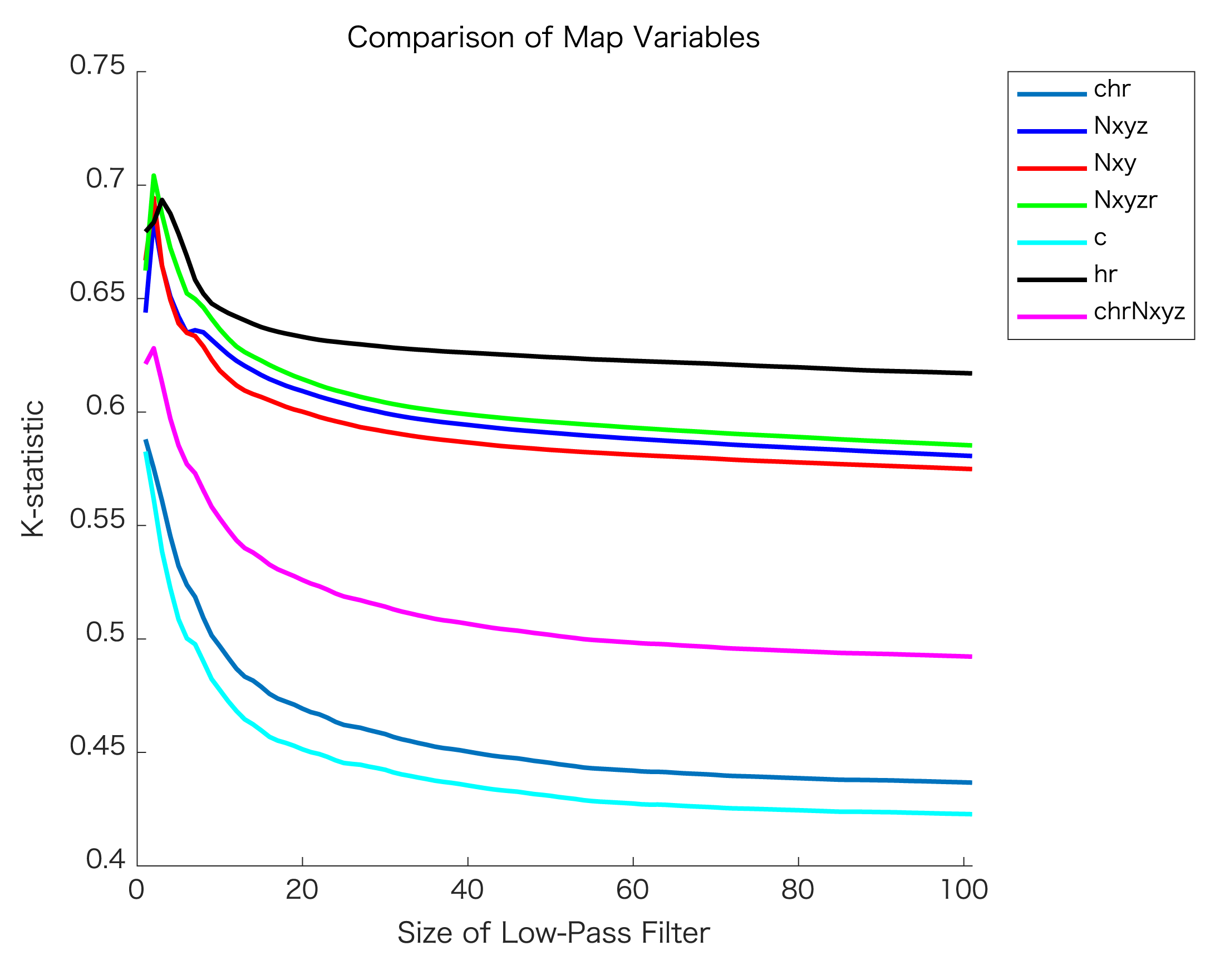

Supplement: S2 Fig — chr, surface curvature, height, and radius; Nxyz, vertex normal; Nxy, two elements of vertex normal; Nxyzr, vertex normal and radius; c, surface curvature; hr, height and radius; chrNxyz, surface curvature, height, radius, and vertex normal. Size of low-pass filter is equal to the number of the set of Fourier coefficients for the analysis. (TIF) [file pcbi.1008436.s003.tif]

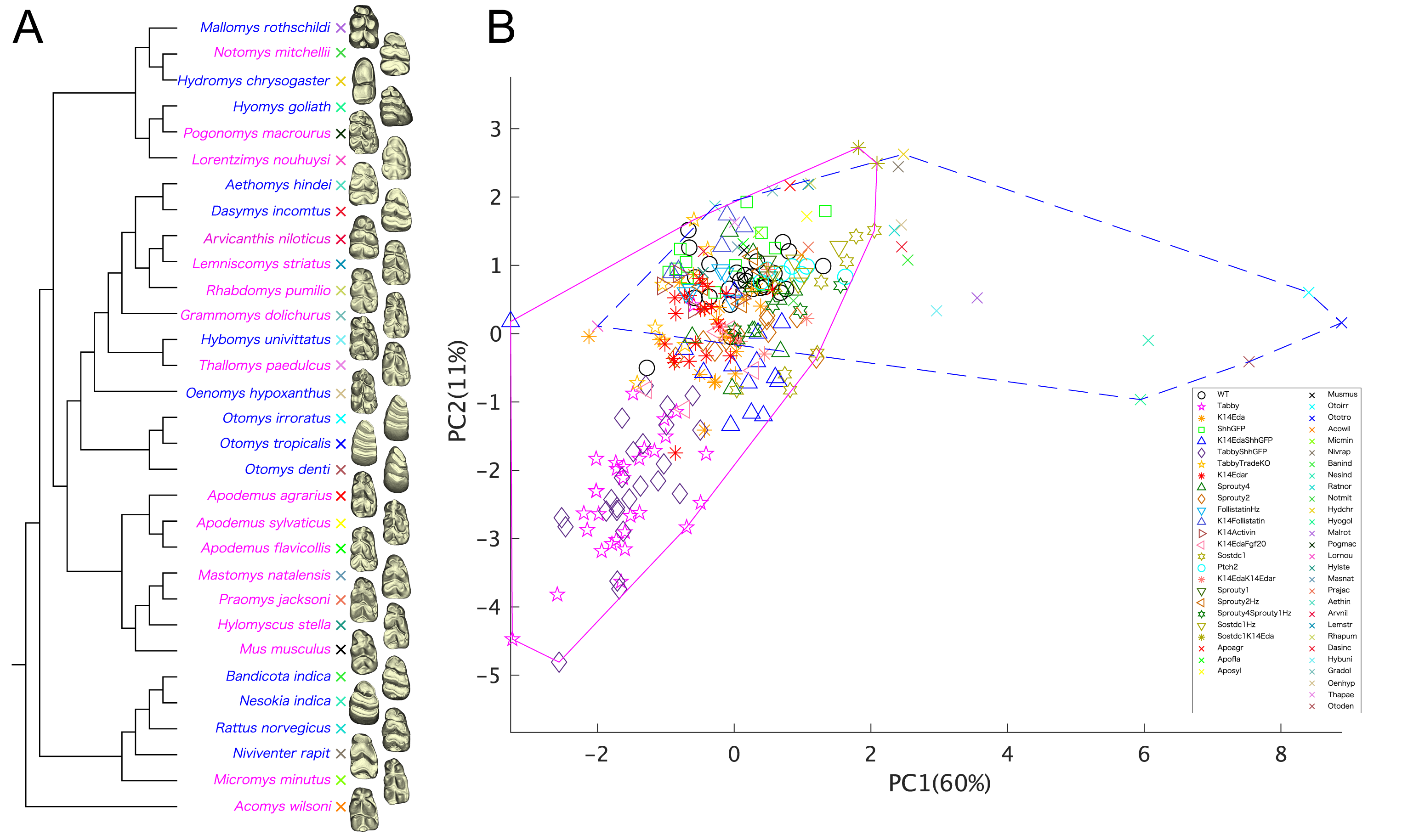

Supplement: S3 Fig — (A) Phylogenetic tree of murines in this study. Taxa locating outside of the mutant range in between-group PC (PC) space are colored with blue otherwise done in magenta. (B) Convex hulls enclose the range of variation of mutants (magenta) and wild murine species (blue) in the first two principal axes of PCA. (TIF) [file pcbi.1008436.s004.tif]
