# Supplementary material for: Mapping molar shapes on signaling pathways
Source: PLoS Comput Biol. 2020 Dec 14;16(12):e1008436. doi: 10.1371/journal.pcbi.1008436 (PMC7735603; doi:10.1371/journal.pcbi.1008436)
Supplement: S1 Table — (DOCX) [file pcbi.1008436.s005.docx]

**S1 Table.** Sample of 22 mutant strains.

| Strain | *N* | References |
| --- | --- | --- |
| WT | 26 | [1] |
| Tabby | 31 | [2, 3] |
| K14Eda | 20 | [4] |
| ShhGFP | 12 | [5] |
| K14EdaShhGFP | 14 | [6] |
| TabbyShhGFP | 24 | [6] |
| TabbyTrade | 7 | [7] |
| K14Edar | 23 | [8, 9] |
| Sprouty4 | 10 | [10] |
| Sprouty2 | 10 | [11] |
| FollistatinHz | 5 | [12, 13] |
| K14Follistatin | 5 | [12, 14] |
| K14Activin | 4 | [15] |
| K14EdaFgf20 | 5 | [4, 16, 17] |
| Sostdc1 | 9 | [18] |
| Ptch2 | 7 | [19] |
| K14EdaK14Edar | 4 | [4, 8, 9] |
| Sprouty1 | 2 | [20] |
| Sprouty2Hz | 4 | [11] |
| Sprouty4Sprouty1Hz | 6 | [10, 20] |
| Sostdc1Hz | 5 | [18] |
| Sostdc1K14Eda | 2 | [4, 18] |

**References**

1. Kangas AT, Evans AR, Thesleff I, Jernvall J. Nonindependence of mammalian dental characters. Nature. 2004;432:211.

2. Laurikkala J, Mikkola M, Mustonen T, Åberg T, Koppinen P, Pispa J, et al. TNF Signaling via the Ligand–Receptor Pair Ectodysplasin and Edar Controls the Function of Epithelial Signaling Centers and Is Regulated by Wnt and Activin during Tooth Organogenesis. Dev Biol. 2001;229(2):443-55.

3. Pispa J, Jung H-S, Jernvall J, Kettunen P, Mustonen T, Tabata MJ, et al. Cusp Patterning Defect in Tabby Mouse Teeth and Its Partial Rescue by FGF. Dev Biol. 1999;216(2):521-34.

4. Mustonen T, Pispa J, Mikkola ML, Pummila M, Kangas AT, Pakkasjärvi L, et al. Stimulation of ectodermal organ development by Ectodysplasin-A1. Dev Biol. 2003;259(1):123-36.

5. Harfe BD, Scherz PJ, Nissim S, Tian H, McMahon AP, Tabin CJ. Evidence for an Expansion-Based Temporal Shh Gradient in Specifying Vertebrate Digit Identities. Cell. 2004;118(4):517-28.

6. Harjunmaa E, Seidel K, Hakkinen T, Renvoise E, Corfe IJ, Kallonen A, et al. Replaying evolutionary transitions from the dental fossil record. Nature. 2014;512(7512):44-8.

7. Kojima T, Morikawa Y, Copeland NG, Gilbert DJ, Jenkins NA, Senba E, et al. TROY, a Newly Identified Member of the Tumor Necrosis Factor Receptor Superfamily, Exhibits a Homology with Edar and Is Expressed in Embryonic Skin and Hair Follicles. J Biol Chem. 2000;275(27):20742-7.

8. Pispa J, Mustonen T, Mikkola ML, Kangas AT, Koppinen P, Lukinmaa PL, et al. Tooth patterning and enamel formation can be manipulated by misexpression of TNF receptor Edar. Dev Dyn. 2004;231(2):432-40.

9. Tucker AS, Headon DJ, Courtney JM, Overbeek P, Sharpe PT. The activation level of the TNF family receptor, Edar, determines cusp number and tooth number during tooth development. Dev Biol. 2004;268(1):185-94.

10. Klein OD, Minowada G, Peterkova R, Kangas A, Yu BD, Lesot H, et al. Sprouty genes control diastema tooth development via bidirectional antagonism of epithelial-mesenchymal FGF signaling. Dev Cell. 2006;11(2):181-90.

11. Shim K, Minowada G, Coling DE, Martin GR. Sprouty2, a Mouse Deafness Gene, Regulates Cell Fate Decisions in the Auditory Sensory Epithelium by Antagonizing FGF Signaling. Dev Cell. 2005;8(4):553-64.

12. Wang XP, Suomalainen M, Jorgez CJ, Matzuk MM, Werner S, Thesleff I. Follistatin regulates enamel patterning in mouse incisors by asymmetrically inhibiting BMP signaling and ameloblast differentiation. Dev Cell. 2004;7(5):719-30.

13. Matzuk MM, Lu N, Vogel H, Sellheyer K, Roop DR, Bradley A. Multiple defects and perinatal death in mice deficient in follistatin. Nature. 1995;374(6520):360-3.

14. Wankell M, Munz B, Hübner G, Hans W, Wolf E, Goppelt A, et al. Impaired wound healing in transgenic mice overexpressing the activin antagonist follistatin in the epidermis. The EMBO Journal. 2001;20(19):5361-72.

15. Antsiferova M, Huber M, Meyer M, Piwko-Czuchra A, Ramadan T, MacLeod AS, et al. Activin enhances skin tumourigenesis and malignant progression by inducing a pro-tumourigenic immune cell response. Nature communications. 2011;2:576-.

16. Huh S-H, Jones J, Warchol ME, Ornitz DM. Differentiation of the Lateral Compartment of the Cochlea Requires a Temporally Restricted FGF20 Signal. PLoS Biol. 2012;10(1):e1001231.

17. Haara O, Harjunmaa E, Lindfors PH, Huh SH, Fliniaux I, Aberg T, et al. Ectodysplasin regulates activator-inhibitor balance in murine tooth development through Fgf20 signaling. Development. 2012;139(17):3189-99.

18. Kassai Y, Munne P, Hotta Y, Penttila E, Kavanagh K, Ohbayashi N, et al. Regulation of mammalian tooth cusp patterning by ectodin. Science. 2005;309(5743):2067-70.

19. Nieuwenhuis E, Motoyama J, Barnfield PC, Yoshikawa Y, Zhang X, Mo R, et al. Mice with a Targeted Mutation of <em>Patched2</em> Are Viable but Develop Alopecia and Epidermal Hyperplasia. Mol Cell Biol. 2006;26(17):6609-22.

20. Basson MA, Akbulut S, Watson-Johnson J, Simon R, Carroll TJ, Shakya R, et al. Sprouty1 Is a Critical Regulator of GDNF/RET-Mediated Kidney Induction. Dev Cell. 2005;8(2):229-39.
