# Supplementary material for: Mapping molar shapes on signaling pathways
Source: PLoS Comput Biol. 2020 Dec 14;16(12):e1008436. doi: 10.1371/journal.pcbi.1008436 (PMC7735603; doi:10.1371/journal.pcbi.1008436)
Supplement: S2 Table — Nxyz, vertex normal; Nxy, two elements of vertex normal; Nxyzr, vertex normal and radius. SVM, Support vector machine. (DOCX) [file pcbi.1008436.s006.docx]

**S2 Table.** Top twenty probabilistic classification models in machine learning for 22 mutant strains. Nxyz, vertex normal; Nxy, two elements of vertex normal; Nxyzr, vertex normal and radius. SVM, Support vector machine.

| Rank | Size of low-pass filter | Classification loss | Combination of maps | Model |
| --- | --- | --- | --- | --- |
| 1 | 6 | 0.209 | Nxyz | SVM |
| 1 | 8 | 0.209 | Nxyz | SVM |
| 1 | 10 | 0.209 | Nxyz | SVM |
| 1 | 6 | 0.209 | Nxyzr | SVM |
| 1 | 10 | 0.209 | Nxyzr | SVM |
| 6 | 20 | 0.213 | Nxyz | SVM |
| 6 | 7 | 0.213 | Nxyzr | SVM |
| 6 | 8 | 0.213 | Nxyzr | SVM |
| 6 | 12 | 0.213 | Nxyzr | SVM |
| 6 | 17 | 0.213 | Nxyzr | SVM |
| 6 | 20 | 0.213 | Nxyzr | SVM |
| 12 | 7 | 0.217 | Nxyz | SVM |
| 12 | 11 | 0.217 | Nxyz | SVM |
| 12 | 17 | 0.217 | Nxyz | SVM |
| 12 | 26 | 0.217 | Nxyz | SVM |
| 12 | 9 | 0.217 | Nxyzr | SVM |
| 12 | 22 | 0.217 | Nxyzr | SVM |
| 12 | 26 | 0.217 | Nxyzr | SVM |
| 12 | 31 | 0.217 | Nxyzr | SVM |
| 12 | 7 | 0.217 | Nxy | SVM |
