# Supplementary material for: Mapping molar shapes on signaling pathways
Source: PLoS Comput Biol. 2020 Dec 14;16(12):e1008436. doi: 10.1371/journal.pcbi.1008436 (PMC7735603; doi:10.1371/journal.pcbi.1008436)
Supplement: S3 Table — *Collection source: §Naturhistorica Riksmuseet Stockholm; †Luonnontieteellinen Museo Helsinki; ‡Institute of Biotechnology, University of Helsinki. Geographical distribution is based on [22,23]. Body mass (mean weight in g) is cited from [24]. Ave. Centroid size is calculated from three-dimensional surface model. (DOCX) [file pcbi.1008436.s007.docx]

**S3 Table.** Sample for wild murine species analysis. *Collection source: ^§^Naturhistorica Riksmuseet Stockholm; ^†^Luonnontieteellinen Museo Helsinki; ^‡^Institute of Biotechnology, University of Helsinki. Geographical distribution is based on [1, 2]. Body mass (mean weight in g) is cited from [3]. Ave. Centroid size is calculated from three-dimensional surface model.

**References**

1. Nowak RM, Walker EP. Walker's Mammals of the World: JHU press; 1999.

2. Fabre P-H, Pagès M, Musser GG, Fitriana YS, Fjeldså J, Jennings A, et al. A new genus of rodent from Wallacea (Rodentia: Muridae: Murinae: Rattini), and its implication for biogeography and Indo-Pacific Rattini systematics. Zoological Journal of the Linnean Society. 2013;169(2):408-47.

3. Wilman H, Belmaker J, Simpson J, de la Rosa C, Rivadeneira MM, Jetz W. EltonTraits 1.0: Species-level foraging attributes of the world's birds and mammals. Ecology. 2014;95(7):2027.
