# Supplementary material for: Mapping molar shapes on signaling pathways
Source: PLoS Comput Biol. 2020 Dec 14;16(12):e1008436. doi: 10.1371/journal.pcbi.1008436 (PMC7735603; doi:10.1371/journal.pcbi.1008436)
Supplement: S4 Table — hr, height and radius; Nxyz, vertex normal; Nxy, two elements of vertex normal; Nxyzr, vertex normal and radius. (DOCX) [file pcbi.1008436.s008.docx]

**S4 Table.** Top twenty models of phylogenetic signal. hr, height and radius; Nxyz, vertex normal; Nxy, two elements of vertex normal; Nxyzr, vertex normal and radius.

| Rank | Size of low-pass filter | K-statistic | *P*-value | Combination of maps |
| --- | --- | --- | --- | --- |
| 1 | 2 | 0.704 | 0.042 | Nxyzr |
| 2 | 2 | 0.694 | 0.047 | Nxy |
| 3 | 3 | 0.693 | 0.024 | hr |
| 4 | 4 | 0.687 | 0.032 | hr |
| 5 | 3 | 0.687 | 0.044 | Nxyzr |
| 6 | 2 | 0.685 | 0.051 | Nxyz |
| 7 | 2 | 0.684 | 0.029 | hr |
| 8 | 1 | 0.679 | 0.051 | hr |
| 9 | 5 | 0.679 | 0.033 | hr |
| 10 | 4 | 0.672 | 0.040 | Nxyzr |
| 11 | 6 | 0.669 | 0.039 | hr |
| 12 | 1 | 0.667 | 0.091 | Nxy |
| 13 | 3 | 0.665 | 0.038 | Nxy |
| 14 | 3 | 0.664 | 0.057 | Nxyz |
| 15 | 1 | 0.662 | 0.076 | Nxyzr |
| 16 | 5 | 0.662 | 0.037 | Nxyzr |
| 17 | 7 | 0.658 | 0.048 | hr |
| 18 | 6 | 0.652 | 0.039 | Nxyzr |
| 19 | 8 | 0.652 | 0.054 | hr |
| 20 | 4 | 0.651 | 0.057 | Nxyz |
