# Supplementary material for: Mapping molar shapes on signaling pathways
Source: PLoS Comput Biol. 2020 Dec 14;16(12):e1008436. doi: 10.1371/journal.pcbi.1008436 (PMC7735603; doi:10.1371/journal.pcbi.1008436)
Supplement: S5 Table — (DOCX) [file pcbi.1008436.s009.docx]

**S5 Table.** Results of phylogenetic generalized least squares (PGLS) on molar shape variation of 31 wild murine species.
